# The Role of Surgical Resection and Liver Transplantation for the Treatment of Intrahepatic Cholangiocarcinoma

**DOI:** 10.3390/jcm10112428

**Published:** 2021-05-30

**Authors:** Guergana Panayotova, Jarot Guerra, James V. Guarrera, Keri E. Lunsford

**Affiliations:** Rutgers New Jersey Medical School, Department of Surgery, Division of Transplant and HPB Surgery, Newark, NJ 07103, USA; gp353@njms.rutgers.edu (G.P.); jjg290@njms.rutgers.edu (J.G.); james.guarrera@njms.rutgers.edu (J.V.G.)

**Keywords:** cholangiocarcinoma, transplant oncology, intrahepatic cholangiocarcinoma, liver transplantation, liver resection, biliary tract malignancy

## Abstract

Intrahepatic cholangiocarcinoma (iCCA) is a rare and complex malignancy of the biliary epithelium. Due to its silent presentation, patients are frequently diagnosed late in their disease course, resulting in poor overall survival. Advances in molecular profiling and targeted therapies have improved medical management, but long-term survival is rarely seen with medical therapy alone. Surgical resection offers a survival advantage, but negative oncologic margins are difficult to achieve, recurrence rates are high, and the need for adequate future liver remnant limits the extent of resection. Advances in neoadjuvant and adjuvant treatments have broadened patient treatment options, and these agents are undergoing active investigation, especially in the setting of advanced, initially unresectable disease. For those who are not able to undergo resection, liver transplantation is emerging as a potential curative therapy in certain cases. Patient selection, favorable tumor biology, and a protocolized, multidisciplinary approach are ultimately necessary for best patient outcomes. This review will discuss the current surgical management of locally advanced, liver-limited intrahepatic cholangiocarcinoma as well as the role of liver transplantation for select patients with background liver disease.

## 1. Introduction

Cholangiocarcinoma (CCA) is the second most common primary liver malignancy following hepatocellular carcinoma (HCC) and comprises approximately 15% of all primary liver cancers and 3% of gastrointestinal malignancies worldwide [1]. CCA is an aggressive malignancy of the epithelial lining of the biliary tract and can be classified based on anatomic location as intrahepatic (iCCA) or extrahepatic (perihilar [pCCA] or distal [dCCA]), with perihilar being the most common [2,3]. Intrahepatic cholangiocarcinoma accounts for 5–10% of CCAs, and its incidence is increasing worldwide [4,5]. Previously staged similar to HCC, iCCA is now recognized as a unique entity with a distinct molecular profile and pattern of disease progression [4].

While the risk factors in the pathogenesis for iCCA are not completely understood, chronic inflammation of the biliary tract appears to be a key contributor [4,6]. Disease can arise in cirrhotic and non-cirrhotic livers. When present in the background of liver-disease, typical patient cancer surveillance allows for earlier identification [5]. Most often, however, early stages are silent, and patients present late in their disease course, resulting in limited treatment options and high morbidity and mortality [3]. In addition to the rarity of these tumors, the variability in patient presentation and specific tumor characteristics present challenges in diagnosis, staging, and management [7]. Diagnosis of localized iCCA requires a combination of imaging, tumor markers, and histological confirmation [1,8]. Distinct pathologic subtypes of iCCA with variable growth patterns and sensitivity to therapy add another layer of complexity to the diagnosis and treatment [1]. Therefore, despite advances in diagnostics and therapeutics, cholangiocarcinoma remains difficult to identify and treat, and with rising incidence worldwide, presents a significant health care burden [8].

A multidisciplinary approach, combining medical and surgical therapy as well as a thorough understanding of tumor biology are key for the optimal management of patients with iCCA. Currently, resection offers the only widely-accepted curative option, but long-term benefits are limited by high recurrence rates. Data from the phase III multicenter BILCAP trial show a median OS of 36 months for surgery alone in patients with biliary tract disease [9]. Due to the locally-aggressive growth pattern and late presentation as well as concerns for adequate future liver remnant (FLR) function and proximity to vital structures, an oncologic resection is only possible for approximately 20% of patients [1,9,10]. For unresectable disease, data from the multicenter phase III ABC-02 trial for treatment of advanced biliary tract cancers demonstrate a median overall patient survival of only 9.6 months, despite best medical therapy with gemcitabine and cisplatin [11,12]. Liver transplantation (LT) is emerging as an alternative treatment for select patients with favorable tumor biology. Transplantation offers the advantage of the widest margins of resection, treatment of underlying liver disease, and obviates the concern for FLR function [12]. However, in this era of persistent donor organ shortage, concerns for cancer recurrence, related mortality, and resource allocation have limited the wide application of LT for the treatment of intrahepatic cholangiocarcinoma.

While cancer is a contraindication to transplant for most organs, liver transplant as a treatment option in the setting of malignancy is evolving. Initial data on LT for biliary tract malignancies showed poor 5-year OS, ranging from 10–30% in different series, and recurrence rates of greater than 50%, with most occurring locally, within two years of transplantation [13,14,15,16,17,18]. Over the past 20 years, refinement in patient and tumor selection criteria and neoadjuvant therapies have improved the success of liver transplant for early-stage perihilar cholangiocarcinoma [19,20,21,22]. The United Network of Organ Sharing (UNOS) and Organ Procurement and Transplantation Network (OPTN) currently grant Model for End-Stage Liver Disease (MELD) exception points to adult patients with unresectable pCCA who meet specific size criteria in the absence of metastatic disease [23,24]. In contrast, iCCA remains a relative contraindication to liver transplantation [5]. However, new data are emerging showing equivalent, if not superior, outcomes for liver transplant versus surgical resection for locally advanced, liver-limited iCCA, prompting renewed interest in this area of transplant oncology. Here, we review the current literature on the surgical management of iCCA as well as the emerging role of liver transplantation in the treatment of this aggressive biliary tract malignancy.

## 2. Background and Risk Factors

Intrahepatic cholangiocarcinoma (iCCA) occurs proximal to the segmental biliary ducts and is classically divided into three subtypes based on growth pattern: mass-forming, periductal infiltrating, and intraductal [1,25]. These correlate with aggressive tumor behavior and patient outcomes, underscoring the variability in tumor biology and need for tailored treatment [26,27]. While chronic inflammation is a risk factor, many occur de novo, without a background of liver disease or symptoms, making timely diagnosis a challenge [28]. Specific risk factors for developing intrahepatic cholangiocarcinoma include environmental exposures, parasitic infections, primary sclerosing cholangitis (PSC), primary biliary stones, and congenital disorders of the biliary system [29,30].

## 3. Preoperative Evaluation and Planning

Surgical resection of gross and microscopic disease, otherwise known as R0 resection, is the only widely accepted curative option. Preoperative evaluation mandates assessment for occult metastatic and advanced locoregional disease. Cross-sectional imaging utilizing computed tomography (CT), magnetic resonance imaging (MRI), or magnetic resonance imaging with cholangiopancreatography (MRI/MRCP) to visualize the ductal and vascular structures as well as delineate extent of disease are standard modalities. The added utility of fluorodeoxyglucose positron emission tomography (FDG-PET) is controversial. FDG-PET is not commonly recommended, especially in the setting of existing CT or MRI imaging and low suspicion for occult metastatic disease [31,32]. Patients with bilateral multifocal disease or with multicentric tumors, when a sufficient tumor margin as well as adequate liver remnant cannot be achieved with surgery, are considered unresectable [33].

Advances in perioperative patient selection, post-operative management, and overall surgical techniques have decreased the morbidity and mortality of surgery for iCCA. Postoperative morbidity after liver resection is reported in up to 43% of patients, with mortality rates as low as 5% in high volume and specialized centers [33]. The most common complications following resection are superficial skin infections (13.1%), intraabdominal abscesses (7.5%), sepsis (6.3%), and bile leak (4%). High volume centers, a multidisciplinary approach, and appropriate patient selection correlate with overall improved outcomes [28,33,34,35,36,37]. In addition to R0 resection, lymph node status is among the most important long-term prognostic factors in iCCA [33,35,38,39,40,41,42]. Additional independent risk factors for survival include serum carcinoembryonic antigen (CEA) and CA 19–9 levels, tumor diameter and number, presence of vascular invasion, and extrahepatic metastases [43,44].

Resectable disease is classically defined as tumors that can be excised completely with adequate liver remnant remaining and negative histologic margins. Macrovascular invasion, bilateral multifocal or multicentric disease, peritoneal disease, and distant lymph node metastasis characterize unresectable tumors [31,45]. Prior to resection, diagnostic laparoscopy can be performed to assess for occult metastatic and/or locoregional disease, which can be seen in up to 30% of resection candidates [46]. The use of intraoperative liver ultrasound can increase the sensitivity of diagnostic laparoscopy and reduces laparotomies in up to one third of patients [46,47,48]. Overall low diagnostic yield as well as cost ineffectiveness limits application of this adjunct, and current recommendations reserve pre-operative laparoscopy for select patients with high-risk features (elevated tumor markers, multi-centric disease, large tumors) to assess for occult metastatic disease [31].

## 4. Surgical Approach

Optimal surgical approach for patients with iCCA varies. Major hepatic resection (≥3 segments) to achieve negative margins is typically performed [49,50]. Less extensive resection can be considered for select patients. Data from a recent multi-center international database analysis of patients undergoing resection of iCCA over a 30-year period suggests minor hepatectomy (two or fewer segments or non-anatomic wedge resection) achieves the same oncologic outcome (OS 37 months vs. 38 months) with the benefit of lower peri-operative morbidity [51,52]. However, when surgical margins are narrow (defined as 1–4 mm), minor resection results in statistically significant inferior recurrence free survival (RFS). In this setting, large, multiple, or bilobar tumors should undergo extended hepatic resection when feasible, for optimal margins and outcomes [53].

Patients can additionally undergo anatomic versus non-anatomic resection of the primary tumor, depending on the location and extent of their disease. In a recent study by Si and colleagues from China, comparing major anatomic (AR) versus non-anatomic resection (NAR), the group noted improved OS (36% vs. 25%) and disease-free survival (DFS) (28% vs. 18%) at 5-years for patients treated with AR [51]. They partially attributed this to more advanced disease and more likely underlying cirrhosis for those ultimately treated with NAR. However, the trend held even when patients underwent propensity score matching. Interestingly, the survival benefit of AR over NAR was noted for stage I and II disease (American Joint Committee on Cancer [AJCC], 8th), but patients with stage III disease had comparable poor outcomes regardless of extent of resection [51]. This suggests tumor biology and extent of disease are important predictors of treatment outcome.

Recently, groups have also explored minimally invasive (MIS) liver resections for cholangiocarcinoma. Overall, studies suggest decreased length of stay, lower incidence of complications, and lower readmission rates compared to open hepatectomies [28,54,55,56,57]. Adoption in the setting of CCA remains limited due to the challenges of performing an extensive resection and regional lymphadenectomy laparoscopically [58]. Therefore, oncologic outcomes data prospectively comparing minimally vs. maximally invasive resection for iCCA directly are limited. When an R0 resection is possible, OS and DFS between laparoscopy and open procedures for iCCA appear comparable [58,59,60]. A metanalysis evaluating 186 patients with iCCA undergoing minimally-invasive vs. open resection showed that the rates of R0 resection were comparable, but lymphadenectomy was performed more frequently in open cases, with higher numbers of harvested nodes. Despite this, DFS and OS were similar between groups [54]. More data are necessary to establish the optimal surgical approach and patient selection for best oncologic as well as overall outcomes.

## 5. Future Liver Remnant and Regenerative Techniques

Assessment of adequate liver function (future liver remnant or FLR function) pre-resection is necessary, especially when an extended hepatic resection is planned and patients present with underlying liver disease. For those with marginal liver remnant, regenerative techniques can be utilized to improve functional capacity. Portal vein embolization/ligation (PVE/PVL) has been shown to significantly increase the FLR volume with no notable difference in achieving R0 resection or subsequent major complication rates in the setting of major hepatic resection for liver malignancy [36,61]. Limitations include timing to regeneration as well as adequate treatment effect, which is limited to 9% vs. 40% hypertrophy in the cirrhotic compared to healthy liver [61,62,63].

Associating liver partition and portal vein ligation for staged hepatectomy or ALPPS procedure may allow staged hepatic resection for large tumors including bilobar tumors, achieving results in six to ten days [62]. While utility in the surgical management of other hepatobiliary malignancies has been reported, there is limited data for ALPPS for iCCA. In a recent multi-center analysis of ALPPS versus chemotherapy in the treatment of locally advanced iCCA, staged resection resulted in an R0 resection in 85.3% of the “unresectable” patients included. Despite a short-term increase in 90-day mortality, when compared to systemic therapy alone, ALPPS resulted in improved 3-year OS (39.6% vs. 11.3%). However, more then 50% of patients recurred, and the majority of recurrences were intrahepatic, occurring within the first year of intervention (median time to recurrence of 9.3 months) [64]. Patients with multiple or larger tumors were more likely to recur. When subgroup analysis by number of lesions was performed, ALPPS resulted in superior survival compared to chemotherapy in patients with one but not multiple lesions [64]. In a single center experience of ALPPS for iCCA from Germany, the authors similarly found feasibility and improved survival for patients undergoing this technique. In this study, patients who were able to complete both stages of the procedure achieved median OS of 4.2 years. All patients were staged T2 or T3 and ranged from N0 to N1 status. Those with nodal metastases did not survive beyond one year following resection. Most patients (82%) recurred within the study follow-up period [65]. While acknowledging the feasibility of this technique to achieve extended resection, in light of high recurrence and limitations when applied to patients with nodal disease or multiple lesions, the role of ALPPS in the management of iCCA remains to be defined. In addition, while achieving expedited results, this technique bears high morbidity and mortality (20% 90-day mortality [66]), which may be exacerbated in patients with iCCA, which bears further evaluation.

## 6. Management of Primary Tumor in the Presence of Metastatic Disease

As iCCA often presents with advanced disease, management of metastases and patient eligibility for resection is an ongoing debate. As expected, patients who develop metastatic disease have a poorer prognosis overall [67]. Evaluating outcomes for patients based on extent of systemic disease, iCCA metastatic to the bone and lungs is associated with a worse prognosis compared to those with liver-limited or nodal disease: 1-year OS (less than 20% vs. 30–40%). When stratified by OS and cancer-specific survival, patients with regional nodal disease benefit from resection of the primary lesion [68]. Liver metastases are of particular interest, as they affect as many as 48% of patients with iCCA [69], can develop in the absence of extrahepatic disease, and affect regional treatment planning [67]. Liver metastases are not specifically recognized in the most recent 8^th^ edition of the AJCC staging for iCCA, but are grouped with multifocal disease (multiple tumors without extrahepatic spread), and in the absence of nodal or extrahepatic disease, are considered T2 disease [4]. However, that definition is inconsistent with reported clinical outcomes. In a recent analysis of a European database of patients with iCCA, Lamarca and colleagues compared patient outcomes between those with early-stage disease (stages I–III) to those with liver metastases [67]. Over a median follow up of 11 months, patients with iCCA with liver metastases had a median OS of 10.97 months, with less than 50% of patients alive at two-years, similar to advanced stage IV disease. Interestingly, the poor prognostic effect of liver metastases is independent of nodal status and is associated with poorly differentiated histology [67,70,71]. This suggests more aggressive disease and speaks to the biologic variability of iCCA [67].

In cases that do proceed to resection, management of regional nodal disease is via regional lymphadenectomy, performed at the time of index operation. Lymph node (LN) metastases are found in as many as 15–45% of patients, in all stages of disease, and correlate with poor oncologic outcomes [27,72,73,74]. For those eligible for primary tumor resection, regional lymphadenectomy has been shown to impart some survival benefit [72]. Standard dissection includes LNs in the hilar, peri-pancreatic, peri-duodenal, and gastro-hepatic areas as well as para-aortic nodes for select cases [70,75,76,77]. The choice of performing a lymphadenectomy has evolved over the past decade [72,74]. While there is a clearer consensus favoring lymphadenectomy in cases of clinically positive regional nodal disease at the time of initial diagnosis, lymphadenectomy for clinically node-negative patients has been an area of controversy. More recent studies have shown an improved overall long-term survival (65% vs. 46% at 5-years) as well as improved survival following recurrence (30% vs. 17% at 5-years) for patients who undergo lymphadenectomy despite clinically negative LNs at the time of diagnosis [72]. Improved local disease control of the perihepatic region as well as increased utilization of adjuvant therapy following operation may explain these results. Therefore, lymph node dissection and harvest for locally advanced, liver-limited iCCA is recommended [74,77]. Most recent staging guidelines suggest a minimum of six harvested LNs for adequate assessment of nodal status at the time of primary tumor resection [78].

## 7. Role of Adjuvant Therapy

While resection offers a survival advantage compared with no therapy or medical therapy alone, recurrence rates are high. Five-year survival is low, at approximately 30%, and correlates with tumor stage, tumor multiplicity, vascular invasion, and lymph node involvement [79,80]. Early recurrence (ER) following surgery is associated with worse patient outcomes and over 20% of patients recur within six months of resection [81]. Those with ER have 5-year OS of only 8.9% compared to 49.8% for those who recur later in their disease course [81]. This is likely due to the more aggressive tumor biology of iCCA and its locally invasive growth pattern compared to other liver tumors, making an R0 resection difficult. Outcomes for R0 and R1 resections (negative versus positive microscopic margins on pathologic evaluation) are comparable for patients with node positive disease. In contrast, in the setting of node negative disease, a positive microscopic margin significantly adversely affects survival [82]. For patients with node positive and/or margin positive disease, the addition of adjuvant chemotherapy, even in resectable disease, may be of benefit and improve OS [79,83,84,85,86,87].

Currently, patients with locally advanced or metastatic cholangiocarcinoma receive gemcitabine-based systemic therapy [79]. Multiple clinical trials have evaluated the utility of additional systemic therapy versus observation alone for patients following resection for biliary tract malignancy (most recently ESPAC-3, PRODIGE-12, and BILCAP) [79]. Results from BILCAP, comparing adjuvant capecitabine to observation following resection of biliary tract cancers, showed a trend for improved median OS (51.1 months vs. 36.4 months) as well as RFS (24.4 months vs. 17.5 months) with the addition of post-operative capecitabine. In this trial, the majority of patients who underwent R0 liver resection (60%) had N0 disease (52%) and had moderately differentiated tumors (49%) [9]. The previously reported PRODIGE-12 trial compared gemcitabine and oxaliplatin versus observation following resection. While there was a trend for improved survival with chemotherapy (30.4 months vs. 18.5 months), this was not significant [88]. When adjusting for confounders such as tumor differentiation and node-positive disease, results from ESPAC-3, which evaluated chemotherapy versus observation following resection of pancreato-biliary tumors, showed a statistically significant survival benefit for patients who received adjuvant therapy [89]. Overall, these data suggest patients would benefit from this adjunct, especially those with high-risk features (multiple lesions, poor differentiation, regional LNs) or positive histologic margins following resection [90]. Based on these results, the American Society of Clinical Oncology and the National Comprehensive Cancer Network currently recommend adjuvant capecitabine for six months following resection of biliary tract malignancy for optimal patient outcomes [91,92].

## 8. Advanced Therapy: Downstaging, Neoadjuvant Treatments, and Targeted Therapy

Only approximately one third of patients with iCCA are eligible for upfront resection at the time of diagnosis. In the absence of resection, long-term survival is rare (less than 30%) [79,93]. In the setting of bilobar disease, vascular invasion, or local invasion of the tumor, locoregional (LRT) plus/minus systemic chemotherapy may provide an alternative treatment or successfully downstage disease, allowing for curative resection. Radioembolization with or without pre-operative chemotherapy for initially unresectable iCCA can achieve comparable OS and RFS to patients who qualify for upfront resection [94]. While there is no standardized treatment regimen at this time, LRT with or without chemotherapy in the setting of locally advanced iCCA is undergoing active study. Combination regimens differ between groups and within the studies, making specific treatment assessment difficult. Despite this, results are promising and show improved OS, with up to 20% of initially unresectable patients going forward with hepatectomy. Out of these, as many as 60% can achieve R0 resection, which portends a more favorable oncologic outcome [94,95]. Patients who ultimately undergo resection have a median OS of 51.9 months [95].

Neoadjuvant gemcitabine has also been employed by some groups prior to resection for locally advanced iCCA, with favorable tumor response and subsequent R0 resection [96]. Combination gemcitabine and platinum-based chemotherapy has been reported to be effective in the management of iCCA with lymph node involvement, allowing for downstaging and subsequent curative resection in some cases [97,98,99]. In a retrospective analysis of 186 patients undergoing upfront resection or systemic chemotherapy followed by surgery for iCCA at a single institution, 53% of patients with initially unresectable disease were able to undergo surgery after neoadjuvant protocol. The majority received gemcitabine plus oxaliplatin combination therapy. Over 50% of patients in both groups received additional adjuvant chemotherapy. Patients treated with chemotherapy followed by resection had greater disease burden and clinically positive nodes at the time of diagnosis. As a result, they were more likely to undergo R1 versus R0 resection. For those who achieved R0 resection, surgical margins were inferior in the chemotherapy plus surgery group versus surgery alone, 0.94 mm vs. 6.6 mm. Despite this, median OS (24.1 vs. 25.7 months) and RFS (14.4 months vs. 11.8 months) was similar between groups, suggesting neoadjuvant chemotherapy can be a critical adjunct for locally advanced, initially unresectable, iCCA [100].

Additional adjuncts in the neoadjuvant setting for iCCA include trans-arterial chemoembolization (TACE), drug-eluting bead chemoembolization, trans-arterial radioembolization (TARE), proton beam therapy (PBT), and hepatic artery infusion pumps [94,101,102,103,104,105]. While data on these therapies are mostly limited to advanced, metastatic iCCA, some reports have suggested successful downstaging of locally advanced disease, resulting in subsequent surgical resection. More studies and trials are necessary to elucidate the exact role and utility of these therapies for the treatment of iCCA, especially in the setting of resectable tumors with the goal to limit recurrence and improve long-term outcomes.

With advanced genomics and next generation sequencing techniques, there is increasing data and interest in target-directed therapies for malignancy. Intrahepatic cholangiocarcinoma harbors several identified recurring mutations (fibroblast growth factor receptor [FGFR], isocitrate dehydrogenase 1 [IDH1], epidermal growth factor receptor [EGFR], tumor protein 53 [TP53], Kirsten rat sarcoma viral oncogene [KRAS], fibroblast growth factor receptor 2 [FGFR2] fusions, and V-raf murine sarcoma viral oncogene homolog B1 [BRAF]), which may lend themselves to these novel therapeutics [79,106,107]. Continued clinical trials investigating targeted therapies, novel immune therapy utilizing programmed cell death 1 (PD1) checkpoint inhibition [108], and more aggressive chemotherapy options such as GAP (gemcitabine, cisplatin, and nab-paclitaxel) [109], may prove of benefit for down-staging in the future, but data are currently limited. Overall, small or solitary nodules, well-differentiated tumors, and tumors without lympho-vascular invasion portend the best outcomes given that these patients are generally candidates for surgical resection [4,79,110]. For patients with unresectable tumors based on size, location, or underlying liver disease, liver transplantation may offer a survival benefit. However, the consideration and selection of patients for liver transplantation for iCCA is still the subject of significant debate.

## 9. Evolving Role of Liver Transplantation

While perihilar CCA has become an accepted indication for liver transplantation over the last decade, iCCA is still considered to be a relative contraindication due to historically poor overall patient survival and recurrence rates as high as 50% [14,15,111]. Recently, however, new data are emerging suggesting transplant may be an option for a select subset of patients with favorable tumor biology. As a result, the International Liver Transplantation Society (ILTS) Transplant Oncology Consensus Conference Working Group has now recognized the potential benefit of liver transplant in select patients with iCCA, provided that it is performed under strict clinical protocols/trials in specialized centers [5,112].

Early studies evaluating liver transplant for iCCA demonstrated dismal outcomes, with 5-year OS and RFS of 18–25% [13,14,113]. In light of these poor results, LT was not adopted into clinical practice, and the majority of outcomes data are from retrospective analyses of patients with incidentally discovered tumors on explants or tumors that were misdiagnosed as HCC and were treated with LT. In 2014, Sapisochin and colleagues published the first multi-center retrospective analysis comparing outcomes for liver transplant in cirrhotic patients with incidental or misdiagnosed iCCA (*n* = 27). These were compared to HCC controls (*n* = 54), matched by etiology of end stage liver disease, the number of tumors, and the size of the largest tumor on pathologic evaluation. Over 60% of patients in each group underwent transarterial chemoembolization; patients receiving neoadjuvant systemic chemotherapy were excluded. Despite a higher risk of recurrence and increased mortality for patients with CCA compared to the HCC controls, subgroup analysis revealed an advantage for those with solitary nodules ≤ 2 cm in diameter. Patients with uninodular lesions ≤ 2 cm had a similar recurrence risk (16% vs. 0%, *p* = 0.4) and similar 5-year actuarial survival (62% vs. 80%, *p* = 0.4) compared to matched HCC controls [114]. These data suggested LT may be a reasonable option for patients with early, small, liver-limited iCCA in the background of liver cirrhosis.

A larger retrospective international multicenter analysis by the same group similarly demonstrated statistically significant improvements in tumor recurrence (18% vs. 61%) and 5-year OS (65% vs. 45%) for patients with “very early” iCCA (single lesions ≤ 2 cm) versus those with advanced disease [115]. A more recent retrospective analysis from the Mayo Clinic, Jacksonville, compared 44 patients with explant diagnosis of iCCA or mixed hepatocholangiocarcinoma (HCC-CCA) to patients with HCC within the Milan criteria. Overall iCCA recurrence and survival rates were inferior. When patients were stratified by pathologic category, those with early CCAs (single lesions, ≤2 cm in diameter, no evidence of vascular invasion) demonstrated similar 5-year survival to the controls (63.6% vs. 70.3%). Vascular invasion and incomplete response to pre-transplant locoregional therapy were independently associated with tumor recurrence [116]. While “very early” iCCA may have improved outcomes, one concern is that identification of such small lesions pre-transplant is difficult. In an attempt to evaluate this size cut-off further, de Martin and colleagues retrospectively compared liver resection versus transplant for larger tumors. Patients with lesions 2–5 cm in diameter demonstrated a 5-year RFS of 74%, similar to those with smaller lesions, suggesting a 2-cm cut-off may be too conservative as a selection criterion, and that more patients may benefit from LT as a curative therapy for iCCA [117].

None of these studies, however, evaluated the role of a multidisciplinary approach to treatment, incorporating neoadjuvant therapy in the treatment paradigm. As evidenced by data on liver resection for iCCA, pre-treatment may select for patients with stable or responsive disease, and improve post-operative outcomes, especially for those with more advanced disease at initial diagnosis. University of California, Los Angeles, was, perhaps, the first to demonstrate the benefits of neoadjuvant chemotherapy when combined with transplant in the management of iCCA. The initial results are difficult to interpret as iCCA and pCCA are considered in combination; nevertheless, patients undergoing liver transplant with adjuvant or neoadjuvant therapy showed improved survival compared to those treated with radical resection plus adjuvant therapy. Tumor size was not a significant predictor of outcome, and most patients had locally advanced disease at the time of diagnosis [118].

The first single center prospective case series of protocolized neoadjuvant chemotherapy followed by liver transplant for iCCA was reported by Houston Methodist and MD Anderson Cancer Center. No specific tumor size cut-off was employed, and the reported median cumulative tumor diameter was 14.2 cm. Following neoadjuvant therapy, patients were considered for transplant if there was sustained tumor radiographic stability for >6 months. Five-year OS for the first six patients was 83.3%, with RFS of 50% [119]. An update by the group in 2019 showed outcomes for three additional patients, with similar survival [120]. Therefore, tumor response to therapy, a potential surrogate for tumor biology, rather than lesion size may be the more important predictor of recurrence and survival.

Despite these promising results, in light of scarce donor resources and the prolonged immunosuppression of patients undergoing transplantation, many still believe the risk of recurrence and complications too great to consider LT for the treatment of iCCA. Few studies have compared resection to transplant directly, and currently, no prospective data are available. In a recent retrospective analysis utilizing the National Cancer Database, Hue and colleagues compared liver transplant to resection for patients with iCCA. Ultimately, 114 patients were compared, matched via propensity score, using patient demographics and tumor characteristics as well as the use of neoadjuvant/adjuvant therapy. Median survival was similar between groups (36 vs. 33 months), even amongst those with very early disease (T0–T2). The authors concluded that liver resection should be first line treatment when feasible, and LT reserved for unresectable cases with multifocal disease and/or background of cirrhosis [121]. These results should be interpreted with caution, however, as granular details on the number of lesions, specific neoadjuvant or adjuvant regimens used, disease response, and tumor recurrence were not provided.

Ultimately, liver transplant in the setting of intrahepatic cholangiocarcinoma is still considered investigational. The ILTS working group consensus statement on liver transplant for cholangiocarcinoma currently recommends liver resection as the treatment of choice for patients with iCCA, with LT reserved for unresectable cases, performed under strict investigational protocols or clinical trials [112]. A prospective trial of LT for very early iCCA in cirrhotic patients (NCT02878473) is recruiting patients with estimated primary completion date in 2026. Wide adoption of LT will likely remain limited pending results from such prospective data, which may definitively establish its role in the management of iCCA. Appropriate patient selection and tumor biology, defined by response to protocolized therapy, will be key to establishing the right patient population who would benefit from LT for this malignancy.

## 10. Conclusions

**Figure 1:**
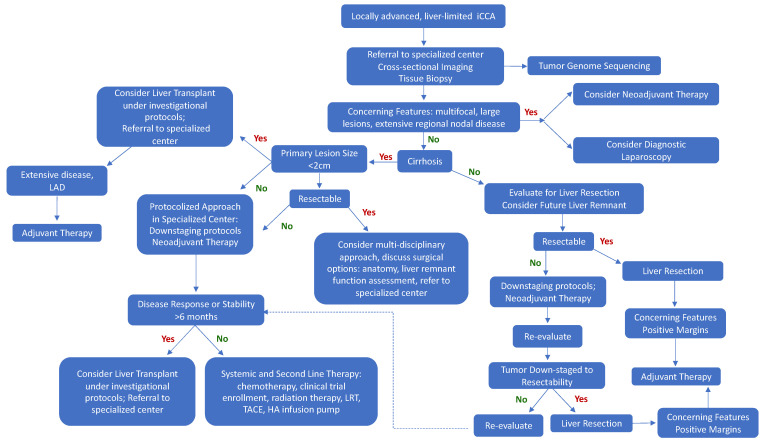
Evaluation and treatment approach for patients with locally advanced, liver-limited intrahepatic cholangiocarcinoma. iCCA: intrahepatic cholangiocarcinoma, LAD: lymphadenopathy, LRT: locoregional therapy, TACE: trans-arterial chemoembolization, HA: hepatic artery.

Cholangiocarcinoma is a rare malignancy of the biliary epithelium and is comprised of a heterogenous mix of tumor types with variable tumor biology and response to therapy. Due to its locally aggressive growth patterns and a propensity for lymphatic spread, patients with iCCA have a dismal prognosis. Improvements in diagnostics, treatment modalities, and tumor surveillance are necessary to optimize patient outcomes. A multidisciplinary, protocolized treatment approach should be considered (Figure 1). Surgical resection with negative microscopic margins is the treatment of choice. Adjuvant therapy may confer some survival benefit, especially for patients with positive margins following resection. In locally advanced disease, neoadjuvant systemic and locoregional therapies may downstage tumors to allow for curative resection. Tumor biopsy with genome sequencing should be considered at the time of diagnosis as novel therapeutics and directed therapies will likely play an increasingly important role in the management of this malignancy. In the setting of locally-advanced, liver-limited disease not amenable for up-front resection, liver transplantation should be considered, especially in the setting of cirrhosis. For small lesions (<2 cm) in the background of liver disease, liver transplant is emerging as superior compared to conventional treatment, and a referral to a specialized center should be considered. In patients with larger lesions (>2 cm), neoadjuvant therapy can be initiated and transplant surgery considered under strict trial protocols. Finally, patients with initially unresectable disease, who respond to neoadjuvant therapy, but are not successfully downstaged to resection, should also be considered for investigational therapy and liver transplant. Early referral to a comprehensive, specialized surgical center with a multidisciplinary treatment approach is key for optimal patient outcomes.
